# Biomarkers in Contrast-Induced Nephropathy: Advances in Early Detection, Risk Assessment, and Prevention Strategies

**DOI:** 10.3390/ijms26072869

**Published:** 2025-03-21

**Authors:** Pei-Hua Lee, Shao Min Huang, Yi-Ching Tsai, Yu-Ting Wang, Fatt Yang Chew

**Affiliations:** 1 Department of Medical Imaging, China Medical University Hospital, Taichung 404, Taiwan; 2 Department of Radiology, School of Medicine, China Medical University, Taichung 404, Taiwan; 3 Department of Medical Education, Show Chwan Memorial Hospital, Changhua 500, Taiwan; 4 Division of Endocrinology, Department of Internal Medicine, China Medical University Hospital, Taichung 404, Taiwan; 5 Department of Pathology, Chung Shan Medical University Hospital, Taichung 402, Taiwan; 6 Department of Pathology, School of Medicine, Chung Shan Medical University, Taichung 402, Taiwan

**Keywords:** contrast-induced nephropathy, iodinated contrast media, biomarkers, kidney injury, risk assessment

## Abstract

Contrast-induced nephropathy (CIN) represents a significant complication associated with the use of iodinated contrast media (ICM), especially in individuals with preexisting renal impairment. The pathophysiology of CIN encompasses oxidative stress, inflammation, endothelial dysfunction, and hemodynamic disturbances, resulting in acute kidney injury (AKI). Early detection is essential for effective management; however, conventional markers like serum creatinine (sCr) and estimated glomerular filtration rate (eGFR) exhibit limitations in sensitivity and timeliness. This review emphasizes the increasing significance of novel biomarkers in enhancing early detection and risk stratification of contrast-induced nephropathy (CIN). Recent advancements in artificial intelligence and computational analytics have improved the predictive capabilities of these biomarkers, enabling personalized risk assessment and precision medicine strategies. Additionally, we discuss mitigation strategies, including hydration protocols, pharmacological interventions, and procedural modifications, aimed at reducing CIN incidence. Incorporating biomarker-driven assessments into clinical decision-making can enhance patient management and outcomes. Future research must prioritize the standardization of biomarker assays, the validation of predictive models across diverse patient populations, and the exploration of novel therapeutic targets. Utilizing advancements in biomarkers and risk mitigation strategies allows clinicians to improve the safety of contrast-enhanced imaging and reduce the likelihood of renal injury.

## 1. Introduction

The utilization of contrast agents in medical imaging is essential for improving the visibility of internal structures; nonetheless, it poses hazards, especially for individuals with renal impairment. Comprehending the components including different types of contrast agents, the incidence and classifications of kidney disease, and the hazards linked to contrast-induced nephropathy (CIN), as well as the function of biomarkers in its therapy is crucial for enhancing patient outcomes in medical imaging treatments.

In this review, we are focusing on iodinated contrast media (ICM) used primarily in computed tomography (CT) scans and angiographic procedures for vascular visualization and tissue differentiation contrasting with gadolinium-based contrast agents, which are frequently employed in magnetic resonance imaging (MRI) and exhibit minimal nephrotoxicity. However, the use of gadolinium-based contrast agents in patients with severe renal impairment remains controversial due to the potential risk of nephrogenic systemic fibrosis, which warrants further discussion. ICM contains iodine, a chemical characterized by a high atomic number and an atomic weight of 127.7, which efficiently absorbs X-rays. ICM can be classified into three categories according to their osmolarities: ionic High-Osmolar Contrast Media (HOCM) possess an osmolality ranging from 1500 to 1800 mOsm/kg, which is five to eight times that of plasma osmolality. Non-ionic Low-OCM (LOCM) possess an osmolality of 600–850 mOsm/kg, which is two to three times that of plasma osmolality, while nonionic iso-OCM (IOCM) have an osmolality of approximately 290 mOsm/kg, comparable to plasma. HOCM exhibit more cytotoxicity in vitro towards proximal tubular cells compared to LOCM or IOCM. High- and low-osmolar iodinated contrast agents exhibit varying levels of nephrotoxicity, with low-osmolar contrast media being more benign [1].

CIN, or Contrast-Induced Acute Kidney Injury (CI-AKI), is characterized by an acute deterioration in renal function occurring within 48 to 72 h following the administration of iodinated contrast media. It is defined by a definitive rise in serum creatinine (sCr) of ≥0.5 mg/dL or a relative increase of ≥25% from baseline levels. CIN is diagnosed following the exclusion of alternative causes of acute kidney injury (AKI) and is linked to procedures such as CT scans and coronary angiography [2,3,4]. It is especially important in patients with pre-existing chronic kidney disease (CKD) or additional risk factors, such as diabetes or advanced age, and may result in heightened morbidity, extended hospitalization, and negative cardiovascular outcomes [3,4,5,6,7,8].

While contrast media are indispensable in modern imaging, their use must be carefully managed to mitigate the risk of CIN, particularly in vulnerable populations. The balance between diagnostic benefits and potential renal risks necessitates a thorough evaluation of each patient’s condition and the implementation of preventive measures. As a result, it is essential to understand the risks of CIN and the role of biomarkers in detection, prevention, and management.

## 2. Prevalence, Pathophysiology, and Risk Factors of CIN

CIN is a common complication, particularly in hospitalized patients undergoing procedures involving contrast media. It is the third leading cause of hospital-acquired AKI. The incidence of CIN ranges from 1–2% in the general population to 1–25% in hospital-acquired AKI cases. While CIN can be reversible, some patients may progress to CKD, with approximately 15% requiring temporary dialysis [3,4,9,10]. Critically ill or elderly patients, especially those with multiple risk factors, are at significantly higher risk, with some studies reporting rates as high as 50% [2,3,5].

Risk factors for CIN encompass patient-related and procedure-related contributors. Key patient-related risk factors include pre-existing kidney disease, particularly CKD, which predisposes individuals to CIN due to compromised renal function. Diabetes mellitus is another significant factor, as it exacerbates renal hypoperfusion and increases vulnerability to nephrotoxic effects. Dehydration further compounds this risk by reducing renal perfusion, while advanced age and comorbidities such as heart failure or hypertension elevate susceptibility to renal injury [10,11].

Procedure-related factors include the volume and frequency of contrast media exposure. The risk of CIN increases with higher volumes of ICM and repeated exposures within a short timeframe, both of which can cumulatively impair renal tissues. Intra-arterial administration of contrast agents, particularly during procedures like coronary angiography, carries a higher risk compared to intravenous administration due to the direct exposure of the kidneys to undiluted contrast media [2]. These combined factors significantly affect the prevalence, severity, and outcomes of CIN, underscoring the need for careful risk assessment and preventive measures in at-risk populations [2,10,11,12,13,14,15,16,17,18].

While many cases of CIN are self-limiting, severe cases can lead to progression to CKD, dialysis dependency, and increased mortality. Patients with pre-existing renal impairment or comorbidities are at a greater risk of poor outcomes. CIN is associated with longer hospital stays, more complicated clinical courses, and elevated risks of cardiovascular events. Following cardiac procedures, the mortality rate increases two- to fivefold in patients developing CIN compared to those without it [2,10].

The likelihood of recovery versus progression to CKD depends on the severity of the initial renal injury and the presence of risk factors such as advanced age, diabetes, or repeated contrast exposure. In less severe cases, in-hospital mortality is about 7.1%, while it rises to 35.7% for patients requiring dialysis [2]. Early identification of at-risk individuals and timely preventive measures can significantly improve outcomes and reduce the likelihood of permanent kidney damage.

As a result, the development of a risk prediction tool for CIN is crucial, as it can help identify patients at high risk for the disorder, especially in populations of advanced age, and the presence of comorbid conditions such as heart failure or hypertension further elevate the risk of CIN [11]. This identification allows for the implementation of preventive strategies, such as intravenous isotonic saline hydration, statins, and other interventions targeting identified risk factors, ultimately aiming to reduce the incidence of CIN.

## 3. Contrast Media and Kidney Function

The infusion of contrast agents, frequently utilized in diagnostic imaging, may result in CIN, a type of AKI. The fundamental processes of CIN remain incompletely elucidated; however, they are thought to involve numerous variables with a complex pathophysiological basis. It encompasses a synthesis of direct nephrotoxic effects from contrast agents, hemodynamic alterations, hypoxia, oxidative stress, apoptosis, inflammation, and immunological responses [2,9,10,11,12,13,14,15,16]. Comprehending these mechanisms, including recognizing risk factors and assessing the incidence and consequences of CIN, is essential for successful preventative and management methods. Renal hemodynamic alterations and nephrotoxic consequences manifest immediately after the intravascular injection of ICM and may endure for hours or even days. The deleterious effects are determined by the inherent characteristics of contrast media, including osmolality and viscosity, in addition to the concentration and volume of contrast media supplied. Typically, CM elicits a fast although non-physiological vasodilation, subsequently succeeded by extended vasoconstriction.

This process may induce renal vasoconstriction, resulting in diminished renal blood flow and ischemia. Ischemia induces the production of reactive oxygen species (ROS), resulting in vascular endothelial damage and tubular injury, mainly attributable to the disparity between vasoconstrictive and vasodilative mediator and intensified by the generation of ROS [2,9,10,12,13,14]. The generation of ROS during contrast media exposure contributes to oxidative stress, damaging renal tubular cells and promoting inflammation and apoptosis. When inflammatory pathways are activated in response to contrast media, this further contributes to renal injury. This involves the recruitment of inflammatory cells and the release of cytokines, which exacerbate tissue damage [10]. Moreover, contrast media elevate blood viscosity, hinder microcirculation, and diminish urine flow rate, hence extending the retention of contrast media in the body and potentially leading to microvascular thrombosis. Collectively, these factors lead to sudden reductions in glomerular filtration rate (GFR) and overall renal function [2,9,10,12,13,14].

## 4. Contrast Media and Biomarkers: Interplay in Kidney Disease

The interplay between contrast agents and biomarkers in kidney disease represents a crucial domain of investigation and therapeutic application, especially in elucidating the processes, identification, and advancement of CIN. Contrast agents significantly impact biomarker levels, affecting their effectiveness for the early detection, diagnosis, and prognosis of CIN. Both traditional biomarkers and emerging novel biomarkers are essential for the development of these processes. This exploration delves into the impact of contrast media on biomarkers, emphasizes their significance in diagnosing CIN, and assesses their potential as prognostic instruments, concentrating on both recognized and newly discovered biomarkers that are influencing the future of nephrology (Table 1).

### 4.1. Traditional Markers of Kidney Function

#### 4.1.1. Serum Creatinine (sCr)

One of the most often utilized indicators in clinical practice, sCr is essential for the identification and treatment of kidney function. It comes from the skeletal muscle’s creatine phosphate and is a metabolic waste product that is mostly eliminated by the kidneys. sCr levels are a valuable metric for evaluating kidney health since they are a functional biomarker that shows glomerular filtration indirectly [19].

In the context of CIN, an elevation in sCr is a crucial diagnostic criterion. A rise in baseline creatinine of ≥0.5 mg/dL or a 25% increase within 48–72 h of exposure to iodinated contrast media is considered CIN [20]. Following angiography of CT with contrast, sCr measurement is now a standard test for evaluating renal function due to its ease of use and the availability of standardized assays.

Despite its utility, sCr has significant limitations. Elevations only happen after significant kidney impairment, when approximately 50% of nephron function damage has already taken place, making it a delayed signal. Furthermore, extrarenal variables including age, gender, muscle mass, and hydration level can affect sCr levels, which could cause misunderstandings. These drawbacks highlight the need for more precise and sensitive biomarkers that can identify kidney damage at an early stage [21,22,23].

sCr is typically sampled through a blood test and analyzed using enzymatic or colorimetric methods. While its role in diagnosing CIN is irreplaceable, healthcare providers can enhance the early detection and management of CIN and eventually improve patient outcomes by being aware of the advantages and disadvantages of sCr [24].

#### 4.1.2. Glomerular Filtration Rate (GFR)

Glomerular filtration rate (GFR) is a fundamental measure of renal function, denoting the volume of blood filtered through the glomeruli per minute. In clinical practice, GFR serves as a critical marker for evaluating renal function and is widely utilized in the diagnosis and staging of CKD and AKI, including CIN [12].

In CIN, contrast media may result in decreased GFR through mechanisms such as medullary hypoxia and direct tubular toxicity. The decline in GFR indicates compromised renal filtration, which may advance to AKI if not rectified. Patients having a baseline estimated glomerular filtration rate (eGFR) of <60 mL/min/1.73 m^2^ are at increased risk of developing CIN after exposure to contrast agents.

Although GFR can be directly measured using exogenous filtration markers like inulin or iohexol, these methods are invasive and not routinely performed. Instead, GFR is commonly estimated (eGFR) using equations like Modification of Diet in Renal Disease (MDRD) or Chronic Kidney Disease Epidemiology Collaboration (CKD-EPI), which rely on sCr or cystatin C (CysC) levels. These equations provide a practical, albeit imperfect, means of assessing renal function.

A notable benefit of GFR is its capacity to identify alterations in renal function at an early stage, frequently prior to the onset of symptoms [25]. Nevertheless, calculated GFR retains the constraints of sCr, including its susceptibility to age, gender, and muscle mass variations. Moreover, direct measurement methods are costly and time-consuming.

Despite its limitations, GFR remains an essential tool in nephrology. The combining of this biomarker with others, such as NGAL, may yield a more thorough evaluation of renal health and enhance the early identification of CIN [26]. By integrating GFR measurements into clinical protocols, healthcare practitioners can more effectively predict and prevent contrast-induced kidney impairment.

#### 4.1.3. Urinary Output (UO)

Urinary output (UO) is a simple yet critical metric in assessing renal function, particularly in acute settings. It indicates the kidneys’ capacity to filter and eliminate fluids, providing a direct indication of renal performance. In the context of CIN, a reduction in UO can signify early renal dysfunction caused by the toxic effects of contrast media. CIN typically involves vasoconstriction and medullary hypoxia, which impair glomerular filtration and tubular function. These processes can lead to oliguria (UO < 0.5 mL/kg/h for 6 h) or even anuria in severe cases. Monitoring urine output in patients having contrast procedures, particularly those with pre-existing risk factors such as chronic kidney disease or diabetes, can serve as an early indicator of renal impairment. On the other hand, urine production of 4150 mL/h in the six hours following the radiological examination has been correlated with diminished incidence of AKI [27]. UO is a non-invasive and cost-effective biomarker. Its real-time nature allows for prompt identification of renal impairment, enabling timely intervention. Owing to certain constraints, it is infrequently employed in clinical practice, as it necessitates consistent urine collection and patient catheterization.

Notwithstanding its constraints, UO continues to be a fundamental element of AKI diagnostic criteria, encompassing the Risk, Injury, Failure, Loss, End-Stage (RIFLE) and Kidney Disease: Improving Global Outcomes (KDIGO) recommendations [19]. The significance of its role in monitoring renal function, particularly in critically ill or high-risk patients, is paramount. Although UO alone may be inadequate for identifying CIN, its application in conjunction with other new biomarkers may improve diagnostic precision and guarantee thorough patient management.

#### 4.1.4. Proteinuria and Microalbuminuria

Proteinuria is defined as an increased concentration of protein in the urine, whereas microalbuminuria refers to albumin levels in the urine that fall within a specific range of 30–300 mg/L. This condition serves as an important indicator of renal impairment and is often associated with CIN. During CIN, tubular injury and alterations in glomerular permeability induced by contrast agents result in the excretion of proteins in the urine that are related to podocyte damage. Proteinuria serves as an early, though non-specific, biomarker for kidney injury [28,29,30].

The straightforward nature of proteinuria testing constitutes a significant advantage. This facilitates regular assessment of renal health, particularly in patients receiving contrast imaging procedures. Nonetheless, its diagnostic utility is constrained by insufficient specificity and vulnerability to external factors, including hydration status and physical activity.

Proteinuria can be assessed through non-invasive techniques, including dipstick tests, spot urine tests, or 24 h urine collections. Elevated levels of urine protein, greater than 150 mg/day, or a random urine protein to creatinine ratio (UPCR) exceeding 15 mg/g may indicate kidney dysfunction and may act as a warning sign for CIN [31]. Proteinuria and microalbuminuria are not exclusive to CIN and may arise from various renal or systemic disorders, including diabetes, cardiovascular disease, hypertension, or inflammation.

Notwithstanding these limitations, proteinuria and microalbuminuria continue to be an important element of a thorough diagnostic strategy for CIN. The combination of specific biomarkers such as NGAL, KIM-1, or L-FABP enhances the accuracy of renal injury assessment [12,19,23]. Integrating these tests into pre- and post-contrast evaluation protocols could facilitate the identification of at-risk patients and inform preventive strategies, thereby decreasing the occurrence of CIN.

### 4.2. Glomerular Filtration and Tubular Dysfunction Biomarkers

#### 4.2.1. Cystatin C (CysC)

CysC has emerged as a promising biomarker for the detection of renal impairment, especially CIN. This low-molecular-weight protein, synthesized by nucleated cells, is readily filtered by the glomerulus and almost entirely reabsorbed in the renal tubules. This renders it extremely responsive to minor fluctuations in GFR, frequently occurring prior to an increase in sCr in identifying renal damage [32].

In CIN, ICM induces vasoconstriction and tubular toxicity, leading to reduced filtration and impaired reabsorption. These pathological alterations lead to increased blood CysC and reduced urine levels, indicating early signs of renal impairment compared to sCr [33]. In contrast to sCr, CysC levels are less influenced by age, gender, or muscle mass, rendering them a more precise indicator of renal function across varied populations [34].

However, CysC is not without limitations. Conditions such as thyroid dysfunction, inflammation, or corticosteroid use might increase its levels, irrespective of renal function, potentially resulting in false positives [35,36,37]. Moreover, its availability in standard clinical environments is not as extensive as that of sCr, hence constraining its utilization.

Despite these challenges, CysC has demonstrated its utility as an effective instrument for the early diagnosis of CIN, especially when a 15% elevation in serum levels within 24–48 h is employed as a diagnostic criterion [38]. Immunoassays, including nephelometry and turbidimetry, are frequently utilized for its quantification. A recent study indicated that CysC at 24 h is the most effective biomarker for diagnosing CIN, although baseline levels of other prevalent biomarkers, including serum IL-18, β-2M, and TNFα, are the most reliable prognostic indicators [39].

#### 4.2.2. Beta-2 Microglobulin (β2M)

Beta-2 microglobulin (β2M) is a small protein integral to the major histocompatibility complex class I molecule. It undergoes free filtration by the glomerulus and is nearly entirely reabsorbed in the proximal tubules of the kidney under standard physiological conditions. Damage to the renal tubules results in reduced reabsorption, leading to increased levels of β2M in both urine and serum. β2M has garnered interest as a potential biomarker for kidney diseases, particularly CIN [40,41,42,43].

Research indicates that serum β2M is a strong predictive biomarker for CIN. Li et al. demonstrated that serum β2M exhibited a notably superior predictive value for CIN compared to sCr, with AUC values of 0.842 (*p* < 0.001) at 24 h and 0.937 (*p* < 0.001) at 48 h, in contrast to sCr’s lower predictive values (AUC 0.691 and 0.908, respectively). These findings indicate that β2M serves as a more effective early predictor of CIN than conventional renal function markers [40].

Serum and urinary β2M levels are quantifiable through immunoassays, including ELISA and nephelometry. Research indicates that baseline β2M levels serve as independent predictors of CIN. Nozue et al. determined that a serum β2M cutoff value exceeding 2.8 mg/L yielded a sensitivity of 75% and a specificity of 80% for predicting CIN. CysC, another renal biomarker, demonstrated a predictive cutoff of >1.26 mg/L, exhibiting comparable sensitivity and specificity. The findings indicate that β2M may serve as an effective early screening tool for identifying patients at risk of CIN prior to contrast procedures [41].

In addition to its predictive capacity for CIN, β2M functions as a prognostic indicator for renal recovery and the progression of disease. Research indicates a significant increase in urinary β2M levels after exposure to contrast media, suggesting tubular damage. In animal models, urinary β2M levels increased by as much as 126-fold following contrast exposure [42]. Similar increases have been noted in human studies; however, statistical significance is still limited [41]. This indicates that urinary β2M is indicative of acute tubular injury, whereas serum β2M may provide a more consistent assessment of renal dysfunction longitudinally.

β2M demonstrates enhanced predictive performance relative to traditional renal function markers such as sCr and eGFR, providing clinicians with a more sensitive and specific instrument for risk assessment, early detection and prognosis. The measurement of β2M in serum and urine enhances diagnostic flexibility, positioning it as a strong candidate for clinical practice integration. Future research must prioritize the validation of optimal cutoff values and the establishment of standardized protocols for β2M measurement to improve its application in routine nephrology and cardiology practices.

#### 4.2.3. Retinol-Binding Protein (RBP)

Retinol-Binding Protein (RBP) is a low-molecular-weight protein synthesized by the liver to transport vitamin A (retinol) in the blood. Under normal conditions, RBP is freely filtered by the glomerulus and almost entirely reabsorbed in the proximal tubules. However, when tubular reabsorption is impaired, as in CIN, RBP levels rise in the urine. RBP is a promising biomarker for detecting proximal tubular injury, making it particularly relevant in the early diagnosis of CIN.

CIN that involves tubular damage may disrupt the reabsorption of RBP in the proximal tubules, leading to increased urinary excretion. Elevated urinary RBP levels can be detected early via urine samples and methods such as ELISA, nephelometry, or turbidimetry, which are widely available and can provide results before significant changes in sCr or GFR, providing a critical diagnostic advantage. However, RBP is not specific to kidney disease and can be elevated in other conditions, such as diabetes, obesity, coronavirus disease, and malnutrition [44,45]. In a randomized controlled study conducted by Umar Sadat et al., urinary RBP levels were found to be elevated in patients undergoing peripheral arterial angiography; however, NAC treatment did not significantly modify this increase, indicating limited nephroprotective effects in this context [46]. In the miniature pig model developed by Junxia Wu et al., exposure to the contrast agent resulted in a significant increase in serum and urinary RBP levels, which peaked within days and persisted at elevated levels for over a week. The findings indicate that RBP is a significant biomarker for diagnosing and prognosing CI-AKI, as it reflects renal tubular injury and recovery after contrast exposure [47]. Both studies highlight the relationship between RBP and CIN. Therefore, by using RBP as part of an early detection strategy, clinicians can identify at-risk patients and take timely steps to mitigate the progression of CIN.

#### 4.2.4. Vitamin D Binding Protein (VDBP)

Vitamin D Binding Protein (VDBP) is a multifunctional glycoprotein that is essential for the transport of vitamin D metabolites and the modulation of immune responses. The role of this biomarker in indicating tubular injury has garnered significant interest [48,49]. Under standard physiological conditions, VDBP undergoes filtration by the glomerulus and subsequent reabsorption by proximal tubular cells. Nonetheless, tubular injury induced by contrast media interferes with this process, resulting in heightened urinary excretion and diminished serum levels of VDBP [50]. Recent studies identify urinary Vitamin D Binding Protein (uVDBP) as a potential biomarker for predicting and evaluating renal injury [51,52,53].

Chaykovska et al. showed that increased uVDBP levels 24 h after contrast administration were significant predictors of the need for dialysis, mortality, and long-term kidney injury up to 90 days post-exposure. The predictive value of uVDBP was validated when adjusted for creatinine excretion, correlating with elevated urinary KIM-1 and baseline plasma creatinine in the affected patients. This study included 314 patients undergoing coronary angiography, which revealed that patients requiring dialysis exhibited significantly higher uVDBP levels (613.07 ± 700.45 ng/mL vs. 113.06 ± 299.61 ng/mL, *p* < 0.001). Additionally, patients who died during follow-up also demonstrated markedly elevated uVDBP levels (522.01 ± 521.86 ng/mL vs. 121.41 ± 324.45 ng/mL, *p* < 0.003). The association of uVDBP with major adverse renal events was independent of traditional CIN risk factors, including anemia, pre-existing renal failure, heart failure, and diabetes, establishing it as a significant early biomarker for CIN and its complications [50]. As a result, urinary VDBP serves as a novel and reliable biomarker for the detection of CIN, providing potential for early diagnosis, risk stratification, and long-term monitoring of renal complications after exposure to contrast media.

### 4.3. Tubular Injury Biomarkers

#### 4.3.1. Kidney Injury Molecule-1 (KIM-1)

Kidney Injury Molecule-1 (KIM-1) serves as a specific biomarker for renal tubular injury and shows considerable potential for the early identification of CIN. This type I transmembrane glycoprotein exhibits minimal expression in healthy kidneys yet shows significant upregulation in proximal tubular cells after nephrotoxic injury [12,19,54,55].

The administration of contrast media in CIN leads to oxidative stress, medullary hypoxia, and epithelial damage. The pathological events induce the release of KIM-1 into the urine, functioning as a non-invasive marker of tubular injury. KIM-1 levels generally increase prior to alterations in sCr as early at 6 h after an angiography procedure, providing an early diagnostic opportunity [56,57,58].

Urinary KIM-1 is beneficial because of its specificity for proximal tubular injury, rendering it a dependable marker for CIN. Nonetheless, it is not solely specific to CIN and may also be elevated in ischemic AKI, CKD, and various other renal conditions. The absence of specificity requires its application alongside additional biomarkers.

KIM-1 detection methods, including ELISA and immunoassays, are relatively simple; however, they have not yet achieved widespread implementation in routine clinical practice. A cutoff value ranging from 0.048 to 6.33 ng/mL has been proposed for predicting CIN, with one study establishing the cutoff as three times the baseline value [59]. Integrating KIM-1 into diagnostic protocols allows clinicians to improve early detection of CIN, facilitating timely interventions and decreasing the risk of progression to more severe renal impairment.

#### 4.3.2. Neutrophil Gelatinase-Associated Lipocalin (NGAL)

Neutrophil Gelatinase-Associated Lipocalin (NGAL) is recognized as an early biomarker for the detection of kidney injury, especially in the context of ischemic and toxic damage [60,61]. NGAL is located at the ascending loop of Henle and the collecting ducts, and it is released from renal tubular cells in response to oxidative stress or inflammation, with levels increasing within hours of contrast exposure. This makes NGAL a more timely indicator of injury than traditional markers such as sCr.

In CIN, contrast media cause tubular damage via mechanisms including medullary hypoxia, the generation of ROS, and direct epithelial toxicity. The processes initiate the release of NGAL, detectable in urine and plasma within 2 to 6 h [19,62]. This swift response offers a significant diagnostic benefit, facilitating earlier intervention and potentially improving clinical outcomes.

NGAL demonstrates high sensitivity to tubular damage yet lacks complete specificity for CIN. Conditions such as sepsis, systemic infections, and chronic kidney disease can also result in elevated levels, potentially leading to false positives. Furthermore, NGAL does not distinguish between the different etiologies of AKI, requiring its application in conjunction with other biomarkers for precise diagnosis [12,19,23].

Notwithstanding these limitations, NGAL continues to be an important instrument in the clinical management of CIN. Plasma or urine levels exceeding 150 ng/mL are highly suggestive of tubular injury [63]. The detection is enhanced through immunoassays, enzyme-linked immunosorbent assay (ELISA), or point-of-care tests, rendering it suitable for routine clinical application. Incorporating NGAL into diagnostic protocols facilitates earlier detection of CIN, allowing for timely interventions and enhanced patient outcomes.

#### 4.3.3. N-Acetyl-β-D-Glucosaminidase (NAG)

N-Acetyl-β-D-Glucosaminidase (NAG) is a lysosomal enzyme secreted by proximal renal tubular cells following injury. This serves as a marker for early tubular damage, thus demonstrating significant relevance in the detection of CIN. NAG typically exists at low concentrations in urine; however, its levels increase markedly when tubular cells sustain damage from oxidative stress or nephrotoxicity induced by ICM [64,65,66,67].

Contrast media during CIN cause hypoxia and free radical generation in the renal medulla, resulting in epithelial damage. This leads to the excretion of NAG in the urine, where its increased activity can be measured. Urinary NAG serves as a non-invasive biomarker that indicates tubular dysfunction at an early stage, frequently before alterations in sCr levels occur [68].

NAG exhibits notable sensitivity to early renal damage. Nonetheless, it is not exclusively associated with CIN and may be elevated in other conditions, including diabetes, hypertension, or general AKI. The absence of specificity restricts its independent diagnostic value and requires its application alongside other biomarkers such as NGAL or KIM-1.

NAG activity is quantified via enzymatic assays employing spectrophotometry or fluorometry and ELISA. Furthermore, uNAG levels reached their peak earlier and exhibited a greater increase compared to sCr levels [68]. Incorporating NAG into diagnostic protocols facilitates earlier detection of CIN, allowing for timely intervention and enhanced patient outcomes.

#### 4.3.4. Liver Fatty Acid-Binding Protein (L-FABP)

Liver Fatty Acid-Binding Protein (L-FABP) serves as an emerging biomarker for the early identification of tubular injury in CIN. This cytoplasmic protein is predominantly expressed in proximal tubular cells, facilitating the binding and transport of fatty acids, while H-type of FABP is localized in distal tubules. L-FABP functions as an antioxidant, providing protection against oxidative stress under normal conditions [19,23].

The administration of ICM in CIN results in medullary hypoxia and an increased production of ROS, which contributes to tubular injury. The pathological changes lead to the release of L-FABP into urine, with elevated levels signifying proximal tubular damage. L-FABP is detectable within hours post-injury up to 48 h, with a peak at 12 h among patients exposed to contrast media who developed CIN [69,70].

L-FABP demonstrates sensitivity to hypoxia-induced injury and can detect tubular damage prior to notable alterations in sCr levels. Nonetheless, its specificity is constrained, as increased urinary L-FABP levels are also detected in CKD, cardiovascular diseases, and various non-CIN conditions [71]. The use of L-FABP alongside other biomarkers is essential for accurate diagnosis.

L-FABP is commonly quantified through immunoassays, particularly ELISAs. A study by Tsukasa Nakamura et al. in 2006 demonstrated that urinary L-FABP levels were significantly greater in patients with CIN compared to patients without CIN and healthy volunteers [72]. As a result, integrating L-FABP into diagnostic workflows enhances early detection and management of CIN, thereby improving clinical outcomes.

#### 4.3.5. α-Glutathione S-Transferase/π-Glutathione S-Transferase (α-GST/π-GST)

α-Glutathione S-Transferase (α-GST) and π-Glutathione S-Transferase (π-GST) are enzymes found in the proximal and distal renal tubules, respectively, and they act as sensitive indicators of tubular injury. In the context of CIN, tubular damage due to oxidative stress and hypoxia leads to the excretion of these enzymes in the urine.

α-GST and π-GST uniquely offer site-specific insights into renal injury. Increased urinary levels of α-GST signify damage to the proximal tubules, whereas heightened π-GST levels indicate injury to the distal tubules. In contrast, elevated serum levels of α-GSTs generally indicate liver damage associated with hepatotoxicity and cannot traverse the glomerular filtration barrier because of their high molecular weight. Measuring urinary α-GST and π-GST offers a diagnostic method for identifying renal injury, as these proteins are specifically expressed in distinct regions of the renal tubules. These are effective instruments for accurately identifying the site of tubular damage in CIN [73]. Like most biomarkers, the detection of α-GST and π-GST necessitates specialized ELISAs, which may not be accessible in all clinical laboratories.

In AKI, a single urinary measurement of π-GST demonstrated greater efficacy than α-GST in predicting the necessity for dialysis or the risk of mortality. Conversely, α-GST exhibited superior performance in patients with stage 1 or 2 AKI. Nonetheless, neither marker demonstrated significant prognostic discrimination in general [74]. However, no significant increase in α-GST was noted after the intravenous administration of the LOCM iohexol in an in vivo study using various rat models of hypertension, diabetes, and nephropathy [75].

#### 4.3.6. Clusterin

Clusterin is a multifunctional glycoprotein involved in cell survival, apoptosis, and tissue remodeling. Clusterin is predominantly located in the cytoplasm of proximal and distal convoluted tubules, where it serves a protective function in kidney injury. Clusterin’s sensitivity to tubular injury and its role in tissue repair processes render it a significant marker for the early detection of renal dysfunction. Increased clusterin levels indicate the degree of injury and the initiation of repair processes in renal tissue. The properties elucidate the kidney’s dynamic response to CIN [76,77,78,79,80,81,82,83,84].

Research indicates that clusterin has anti-apoptotic properties, contributing to cellular protection and regeneration in instances of nephrotoxicity, ischemia, and post-surgical renal injury. In multiple nephrotoxic models, such as cisplatin-induced injury, urinary clusterin levels have demonstrated superior efficacy compared to traditional biomarkers like BUN and sCr in identifying proximal tubular damage prior to the onset of functional decline. Ischemia/reperfusion models demonstrate that clusterin deficiency exacerbates renal inflammation and fibrosis, underscoring its essential role in kidney repair and proliferation. In CIN, a study conducted by YH Deng et al. investigated CIN in a rat model and identified clusterin among 604 distinct proteins implicated in the pathogenesis of CI-AKI. The study utilized proteomic analysis to identify significant pathways, such as the complement and coagulation cascade, PPAR signaling, ferroptosis, and proximal tubule bicarbonate reclamation. Clusterin was validated in conjunction with 16 candidate proteins, which included five novel proteins (Serpina1, Apoa1, F2, Plg, and Hrg) associated with acute response and fibrinolysis. The findings indicate that clusterin and related pathways may significantly contribute to the early diagnosis and prognosis of CI-AKI [83]. Yi Da et al. conducted a randomized clinical trial to assess clusterin as a potential biomarker for drug-induced AKI [84]. The research examined serial urine samples from patients undergoing nephrotoxic treatment, comparing biomarker levels between individuals who developed AKI and matched non-AKI controls. Clusterin, in conjunction with beta-2-microglobulin, KIM-1, MCP1, and CysC, exhibited significantly elevated levels in AKI cases 1–3 days prior to onset. Clusterin exhibited the highest predictive accuracy at 86% and peaked earliest among the biomarkers, indicating its potential for early detection of nephrotoxicity. The findings suggest that serial monitoring of clusterin may offer a significant lead time for the detection of kidney injury, especially in cases of contrast-induced and drug-related nephrotoxicity.

Clusterin can be quantified through ELISA or immunoassays, which are widely available in numerous research laboratories. Although clusterin presents certain advantages, its clinical utility is constrained by insufficient specificity. Increased levels are noted in various conditions, including cancer, cardiovascular disease, and systemic inflammation, potentially resulting in diagnostic ambiguity [85,86,87]. Additionally, data regarding the role of clusterin as an independent biomarker for CIN are limited, and standardized cutoff values have yet to be established. Additional research is required to determine its clinical utility in the prognosis and treatment of CIN.

### 4.4. Inflammatory and Oxidative Stress Biomarkers

#### 4.4.1. Interleukin-18 (IL-18)

Interleukin-18 (IL-18) is a cytokine of the IL-1 superfamily that functions as a pro-inflammatory cytokine, playing a critical role in immune regulation and tissue inflammation. This biomarker is indicative of tubular inflammation and functions as an early marker for acute kidney injury involving significant tubular damage due to oxidative stress and hypoxia, resulting in the release of IL-18 from proximal tubular epithelial cells [12,19].

In the context of CIN, the use of contrast media leads to the production of ROS and medullary hypoxia, subsequently activating the inflammatory pathway. The secretion of IL-18 is detectable in both urine and blood samples. Increased urinary IL-18 concentrations correlate with the extent of tubular injury, establishing it as a significant biomarker for early diagnosis. IL-18 exhibits a notable specificity for acute tubular inflammation. In contrast to sCr, which is elevated later in the progression of kidney injury, IL-18 levels increase early, frequently within hours of injury [88,89,90,91,92]. A study by Zdziechowska, M. et al. examined patients who underwent planned or emergency coronary angiography and received contrast agents. Serum IL-18 levels were analyzed at baseline, 24 h, and 72 h post-procedure. The study showed that IL-18 levels were elevated in patients with CIN, approaching statistical significance after 24 h. Nonetheless, serum IL-18 levels showed a decline at 72 h, indicating its potential ineffectiveness as a biomarker for CIN [89].

IL-18 is commonly quantified through ELISA and Bioplex, where a urinary concentration with a cutoff of >25% at 24 h after usage of contrast agent is indicative of CIN.

#### 4.4.2. C-Reactive Protein (CRP) and High-Sensitivity CRP (hs-CRP)

C-reactive protein (CRP) is an acute-phase protein synthesized by the liver in response to inflammation, predominantly influenced by interleukin-6 (IL-6). High-sensitivity C-Reactive Protein (hs-CRP) represents an enhanced testing method for CRP, allowing for the detection of low levels of systemic inflammation with increased accuracy. They are essential in the immune response by attaching to apoptotic cells or bacterial surfaces, thereby facilitating macrophage phagocytosis. Inflammation is recognized as a risk factor for CIN, leading to extensive research on CRP as a predictive biomarker. Elevated CRP levels contribute to acute kidney injury by impairing the regeneration of tubular epithelial cells through the inhibition of the CDK2/cyclin E pathway mediated by Smad3 [93,94,95].

Research consistently indicates a notable correlation between elevated CRP levels and the incidence of CIN. Gao et al. conducted an analysis of 4522 patients undergoing PCI and determined that patients with CRP levels exceeding 3.0 mg/L exhibited a significantly elevated risk of CIN, even after controlling for baseline variables. Hs-CRP serves as a more accurate indicator and has been demonstrated to predict the risk of CIN [96]. Lazaros et al. created the Athens CIN Score, which integrates hs-CRP, age, GFR, and ejection fraction (EF), showing significant accuracy in predicting CIN [97]. Kim et al. identified that elevated pre-procedural hs-CRP levels exceeding 5 mg/dL serve as a significant and independent predictor of CIN in patients undergoing neurointervention [98].

Both CRP and hs-CRP are significant inflammatory biomarkers for assessing the risk of CIN, and they are measurable through high-sensitivity immunoassays or turbidimetry. The elevation of CRP prior to contrast administration, specifically CRP levels exceeding 3.0 mg/L and hs-CRP levels above 5 mg/dL, is significantly correlated with a higher incidence of CIN. Incorporating CRP into risk prediction models such as the Athens CIN Score could improve early identification and preventive measures for patients at risk.

#### 4.4.3. Neutrophil-to-Lymphocyte Ratio (NLR)

The Neutrophil-to-Lymphocyte Ratio (NLR) serves as an emerging biomarker for systemic inflammation and has been extensively researched for its prognostic significance in cardiovascular diseases and renal dysfunction. Recent evidence underscores its importance in CIN, a significant complication arising from contrast medium exposure during diagnostic and interventional procedures, including PCI and CA. Inflammation is pivotal in the pathogenesis of CIN. The NLR, indicating the balance between pro-inflammatory neutrophils and anti-inflammatory lymphocytes, is a significant predictor of renal injury and CIN progression [99,100,101,102,103,104,105,106,107,108].

The NLR is an easily obtainable and economical parameter derived from a standard complete blood count (CBC), calculated by dividing the neutrophil count by the lymphocyte count. Multiple studies have established cutoff values that signify a heightened risk of CIN. Kurtul et al. found that an NLR of 3.46 or higher was significantly linked to CIN in patients with non-ST-elevation acute coronary syndrome undergoing PCI (OR = 2.631, *p* = 0.022) [102]. Fangfang Zhou et al. similarly identified that an NLR greater than 2.844 serves as an independent risk factor for CIN (OR = 2.304, *p* < 0.001), thereby reinforcing its predictive significance [106]. A meta-analysis conducted by He T et al. encompassing 31 studies established a significant correlation between increased NLR and the development of CIN, reporting a pooled sensitivity of 74.02% and specificity of 60.58%. The analysis indicated that NLR may function as a dependable early detection marker for CIN, enabling prompt intervention and nephroprotective measures [105].

The mechanistic relationship between NLR and CIN is largely influenced by systemic inflammation and oxidative stress, which leads to renal endothelial dysfunction and tubular injury after contrast exposure. Elevated neutrophils release pro-inflammatory cytokines, including IL-6 and TNF-α, which exacerbate renal damage. Concurrently, lymphopenia indicates a weakened immune response, diminishing anti-inflammatory regulation and heightening susceptibility to CIN. Kaya A et al. conducted a study showing that patients with elevated NLR exhibited significantly higher sCr levels following contrast exposure, especially among those with diabetes mellitus, CKD, and reduced left ventricular ejection fraction. Additionally, machine learning models that integrate NLR into predictive algorithms, including the Naïve Bayes (NB) model, attained an AUC of 0.774, establishing this as the most precise model for predicting CIN [100].

Due to its significant predictive and prognostic value, the NLR ought to be incorporated into models for assessing the risk of CIN to facilitate the early identification of high-risk patients. Given the narrow therapeutic window of CIN, employing NLR as an early biomarker could assist clinicians in executing timely interventions and nephroprotective strategies, thereby decreasing the incidence and severity of CIN in cardiovascular patients undergoing contrast-based procedures. Additional prospective studies and extensive clinical trials are necessary to confirm NLR as a standardized biomarker for the prevention and management of CIN.

#### 4.4.4. Red Cell Distribution Width (RDW)

Red cell distribution width (RDW) quantifies the variability in the dimensions of circulating red blood cells and is frequently utilized in standard CBC assessments. This phenomenon indicates erythrocyte heterogeneity and is affected by factors including inflammation, oxidative stress, and compromised erythropoiesis. RDW is evaluated using automated hematology analyzers, and the results are generally reported as a percentage. An elevated RDW is linked to multiple pathological conditions, such as cardiovascular diseases and renal impairment [109,110,111,112,113,114].

Numerous studies indicate a notable correlation between increased RDW and CIN. Kurtul et al. demonstrated that elevated RDW levels independently predict CIN in patients with ACS undergoing PCI [110]. Akkoyun et al. found that elevated RDW serves as an independent predictor of CIN in patients with STEMI undergoing PCI, with a cutoff value of 16.9% [111]. Sun et al. expanded on these findings by assessing the red blood cell distribution width-to-albumin ratio (RAR), establishing that RAR serves as an independent risk factor for CIN in elderly STEMI patients undergoing emergency PCI, with an optimal cutoff value of 3.64 [112]. RDW possesses prognostic significance in the prediction of CIN. Zou et al. demonstrated that an increased RDW following cardiac surgery correlates with a higher incidence and severity of AKI, as well as elevated hospital mortality rates [109]. Mizuno et al. demonstrated that the combination of RDW and the Mehran risk score (MRS) enhances predictive accuracy for CIN in patients with STEMI [113]. Li et al. demonstrated that RDW showed effective discrimination for CIN following enhanced CT, achieving an AUC of 0.803 [114].

The association of RDW with inflammation, oxidative stress, and endothelial dysfunction likely underpins its role in predicting and prognosing CIN, all of which are factors contributing to kidney injury. Increased RDW may signify underlying systemic stress, compromised erythropoiesis, and diminished renal reserve, rendering it a useful biomarker for early risk assessment. Due to its straightforward measurement and routine availability in blood tests, RDW may function as an effective parameter for identifying high-risk patients and informing preventive strategies against CIN.

### 4.5. Cell Stress and Apoptosis Biomarkers

#### 4.5.1. G1 Cell Cycle Arrest Proteins—Insulin-like Growth Factor-Binding Protein 7 (IGFBP-7) and Tissue Inhibitor of Metalloproteinases-2 (TIMP-2)

Insulin-like Growth Factor-Binding Protein 7 (IGFBP-7) and Tissue Inhibitor of Metalloproteinases-2 (TIMP-2) are biomarkers that have received considerable focus for their synergistic role in identifying early renal tubular stress and injury, especially in patients with CIN. Both proteins play a role in the regulation of G1 cell cycle arrest, an essential protective mechanism activated by renal tubular cells in response to stress conditions [12,19,115].

In the context of CIN, oxidative stress, hypoxia, and inflammation impair tubular cell function, resulting in injury and cell cycle arrest at the G1 phase. This arrest serves to inhibit the proliferation of damaged cells. Proximal tubular cells release IGFBP-7 and TIMP-2 under stress conditions, indicating early injury and maladaptive cellular responses. The product of their concentrations, represented as [IGFBP-7] × [TIMP-2], is a significant indicator of tubular cell stress and a predictive marker for AKI, including CIN.

The combination of IGFBP-7 and TIMP-2 is notably effective in identifying subclinical injury prior to observable alterations in functional markers such as sCr or GFR. A cutoff value exceeding 0.3 (ng/mL)^2^/1000 for the [IGFBP-7] × [TIMP-2] product is established for identifying patients at risk of CIN, facilitating early interventions [115].

Although IGFBP-7 and TIMP-2 offer certain benefits, they lack complete specificity for CIN. Both biomarkers may exhibit elevations in various forms of AKI, including those resulting from sepsis, ischemia, or systemic inflammation. The expense associated with assays such as NephroCheck^®^ may restrict their regular use in clinical settings. External factors, including inflammation and comorbid conditions, may influence levels, contributing to diagnostic variability [115].

The IGFBP-7 × TIMP-2 product signifies notable progress in the field of nephrology. Incorporating this biomarker combination into diagnostic protocols enables clinicians to identify at-risk patients earlier and implement preventive measures, including hydration therapy and contrast volume minimization. This method may substantially enhance outcomes by decreasing the frequency and severity of contrast-induced kidney injury [115,116,117].

#### 4.5.2. Dickkopf-3 (DKK3)

Dickkopf-3 (DKK3) is a glycoprotein induced by stress and sourced from renal tubular epithelial cells, showing potential as a biomarker for predicting AKI and the progression of CKD. DKK3 is involved in renal fibrosis through the modulation of the Wnt/β-catenin signaling pathway and is re-expressed in response to pathological stress in the kidney. The urinary DKK3 (uDKK3), assessed through the DKK3-to-creatinine ratio (uDKK3/uCr), has been thoroughly investigated in multiple nephropathies, particularly CIN, which frequently occurs after exposure to iodinated contrast media during CA and PCI [118,119,120,121,122].

The quantification of uDKK3 is conducted through ELISA utilizing spot urine samples. Increased pre-procedural uDKK3/uCr levels are significantly correlated with the risk of CIN development, even among patients without apparent CKD. Seibert et al. conducted a study revealing that patients with CIN had an uDKK3/uCr ratio 3.8 times greater than those without CIN (AUC: 0.61), suggesting a cutoff value of 1.7 pg/mg creatinine [119]. Roscigno et al. conducted a study that confirmed uDKK3/uCr as a predictor of CIN, identifying a threshold of ≥491 pg/mg as the most effective for recognizing at-risk patients [120]. Additionally, urinary DKK3 enhances risk stratification for CIN and acts as a prognostic marker for ongoing kidney dysfunction, with levels ≥322 pg/mg indicating a likelihood of long-term renal impairment. The predictive accuracy of uDKK3/uCr enhances established CIN risk models, such as the Mehran, Gurm, and National Cardiovascular Data Registry (NCDR) scores, thereby improving clinical decision-making.

The upregulation of DKK3 indicates renal tubular stress and susceptibility to fibrosis, implying that patients with increased uDKK3/uCr levels are more vulnerable to contrast-induced tubular injury. Due to its potential in early risk assessment, uDKK3/uCr may be incorporated into clinical workflows to inform preventive strategies, including contrast minimization and customized hydration protocols. Additional large-scale studies are necessary to confirm these findings and investigate potential therapeutic interventions aimed at DKK3 to reduce CIN and the progression of CKD.

#### 4.5.3. Growth Differentiation Factor-15 (GDF-15)

Growth Differentiation Factor-15 (GDF-15) is a cytokine induced by stress and is a member of the transforming growth factor-β (TGF-β) superfamily. Under typical physiological conditions, its expression is minimal; however, it is markedly upregulated in reaction to inflammation, oxidative stress, hypoxia, and endothelial dysfunction. GDF-15 is extensively expressed in cardiac tissue, adipocytes, vascular endothelial cells, and renal tubular cells, contributing to tissue protection and repair via signaling pathways including JNK, EGFR, PI3K, and AKT. Researchers have investigated its role as a potential biomarker for CIN, CKD, and cardiovascular complications, given its involvement in cellular stress responses [123,124,125,126,127,128].

GDF-15 levels are commonly assessed through ELISA or commercial immunoassay kits utilizing serum or plasma samples. Numerous studies have shown its capability in predicting CIN. Sun L. et al. found that GDF-15 levels were significantly increased in patients who experienced CIN after PCI, with an AUC of 0.744, suggesting moderate predictive capability [128]. Furthermore, the integration of GDF-15 with sCr enhanced the predictive accuracy for CIN relative to the use of sCr in isolation. Sun L. et al. demonstrated that GDF-15 levels were significantly elevated in patients with CIN PCI for AMI (1232 ± 366.6 ng/L vs. 939.20 ± 309.6 ng/L, *p* < 0.001). Furthermore, patients in the highest quartile (Q4) of GDF-15 exhibited a 3.57-fold increased risk of CIN compared to those in lower quartiles.

In addition to its role in diagnosis, GDF-15 functions as a prognostic biomarker for the deterioration of renal function and the prediction of long-term negative outcomes [124]. Charlotte Delrue et al. noted that GDF-15 downregulates inflammation and enhances nephroprotective factors such as Klotho. However, consistently elevated levels of GDF-15 correlate with the progression of CKD, accelerated decline in renal function, and poorer prognosis across various renal disorders [123]. Shiori Kobayashi et al. identified GDF-15 as a significant predictor of 3-year decline in renal function, demonstrating an odds ratio (OR) of 1.072 for each 100 pg/mL increase [124]. Xue Bao et al. found that patients in the highest quartile of GDF-15 exhibited a 2.37-fold increased risk of CKD. Furthermore, each one standard deviation increase in GDF-15 was associated with a decline in eGFR of 0.97 mL/min/1.73 m^2^ (*p* < 0.001) [125].

The sensitivity and specificity of GDF-15 in predicting contrast-induced nephropathy differ among various studies. The AUC for GDF-15 alone was 0.744, which was lower than that of sCr; however, its combination with sCr significantly enhanced predictive accuracy. Sun L. et al. reported that in patients with AMI undergoing PCI, the AUC for GDF-15 was 0.793 (95% CI: 0.729–0.856, *p* < 0.001), surpassing the AUC of the ACEF risk score, which was 0.708. The net reclassification improvement (NRI) of GDF-15 compared to ACEF was 0.32 (95% CI: 0.123–0.518, *p* = 0.001), indicating enhanced predictive value in risk stratification.

Elevated GDF-15 levels are linked to renal prognosis, all-cause mortality, and major adverse clinical events. Sun L. et al. established that elevated GDF-15 levels were associated with an 8.43-fold increase in mortality risk and a 3.56-fold increase in the risk of major adverse cardiovascular events (*p* < 0.001). Additionally, CIN was found to be independently linked to higher mortality and MACE in AMI patients (HR: 3.535, *p* = 0.029 and HR: 5.154, *p* < 0.001, respectively), underscoring the significance of GDF-15 as a diagnostic and prognostic marker in CIN.

GDF-15 serves as a promising biomarker for early detection, risk stratification, and prognosis in CIN. Although its AUC is inferior to that of sCr alone, the integration with conventional renal biomarkers improves predictive accuracy. Furthermore, GDF-15 serves as a predictor for long-term renal dysfunction, chronic kidney disease progression, and mortality, thereby enhancing its utility in clinical decision-making. Additional large-scale prospective studies are required to confirm GDF-15 as a standardized biomarker for routine assessment and management of CIN risk.

### 4.6. Vascular and Endothelial Dysfunction Biomarkers

#### 4.6.1. Vascular Endothelial Growth Factor (VEGF)

Vascular Endothelial Growth Factor (VEGF) is a multifunctional signaling protein that is essential for vascular health, particularly in facilitating angiogenesis and sustaining vascular permeability. VEGF exhibits dual function: it facilitates renal microvascular repair, yet excessive VEGF activity may worsen endothelial dysfunction and tubular injury. VEGF is predominantly expressed in glomerular podocytes and tubular epithelial cells within the kidney, whereas its receptors are mainly found on preglomerular, glomerular, and peritubular endothelial cells. VEGF is believed to increase capillary permeability and facilitates the repair of injured microvasculature when there is renal injury induced by contrast agents. Dysregulated VEGF activity can disrupt vascular homeostasis, resulting in inflammation, capillary leakage, and additional renal injury. VEGF is recognized for its involvement in renal function; however, its specific role in normal kidney physiology is not well understood [129,130,131].

VEGF levels may be quantified in serum, urine, or renal tissue through ELISA or immunohistochemical techniques. Elevated VEGF levels indicate renal microvascular stress; however, their diagnostic utility in CIN is constrained by insufficient current clinical data on CIN. Nonetheless, experimental research indicates possible involvement of VEGF in renal damage. A study in rats indicated that VEGF protein levels were significantly increased in kidney tissue after the administration of contrast media, which correlated with the severity of kidney injury. Paricalcitol pretreatment mitigated kidney damage by reducing VEGF levels, indicating a potential preventive strategy for CI-AKI [132]. Wang Y et al. examined renal alterations in a diabetic rabbit model of CI-AKI, evaluating HIF-1α and VEGF expression at various time points post-contrast administration. In diabetic rabbits subjected to contrast agents, sCr and blood urea nitrogen (BUN) reached their maximum levels on day 3, while healthy rabbits exhibited only minor, statistically insignificant elevations in these markers following iohexol administration [133]. Notwithstanding these limitations, VEGF offers significant insights into the renal microvascular response during CIN. The incorporation of this biomarker alongside others could improve diagnostic precision and assist clinicians in comprehending the dynamic mechanisms associated with renal injury. Further investigation is required to examine the therapeutic modulation of VEGF pathways to alleviate CIN-related endothelial dysfunction and enhance patient outcomes.

#### 4.6.2. Osteopontin

Osteopontin (OPN) is a multifunctional glycoprotein involved in immune regulation, cell adhesion, and tissue remodeling. The expression of this biomarker is increased in response to tubular injury resulting from oxidative stress and hypoxia, indicating its potential as a biomarker for CIN. Increased levels of OPN are associated with inflammation, tubular damage, and fibrosis, establishing this as an early indicator of kidney injury [134,135,136].

Multiple studies indicate a positive correlation between increased OPN levels and the risk of AKI, underscoring its potential utility as a diagnostic and prognostic marker [137,138,139]. In a clinical study utilizing proteomics-supported biomarker models, OPN emerged as a significant predictor of procedural AKI, along with diabetes history, the blood urea nitrogen-to-creatinine ratio, C-reactive protein, and additional factors. The incorporation of OPN into a CI-AKI risk model markedly enhanced predictive accuracy, elevating the c-statistic from 0.69 to 0.73 (*p* < 0.001) and improving risk stratification for subsequent cardiorenal outcomes and CKD progression [137].

Furthermore, research conducted within the CASABLANCA cohort demonstrated that OPN, in conjunction with CysC, markedly enhanced the prediction of CI-AKI and long-term negative outcomes, including nonprocedural AKI, CKD progression, myocardial infarction, and all-cause mortality [138,139]. The findings indicate that OPN is integral to the mechanisms of kidney injury, primarily through its role in inflammation, fibrosis, and cellular stress responses. The predictive value of this factor in post-cardiac arrest patient outcomes underscores its significance in assessing risks associated with renal and cardiovascular diseases. OPN may function as a significant biomarker alongside other markers for the early detection, prognosis, and risk stratification of CI-AKI, contributing to the prevention and management of CIN.

OPN can be quantified in urine, serum, or renal tissue through the application of ELISA or Western blot methodologies. While its sensitivity to tubular stress presents a benefit, OPN lacks specificity for CIN. It is increased in several other conditions, including chronic kidney disease, diabetes, and cardiovascular disorders. Furthermore, the absence of a standardized cutoff for OPN levels in predicting CIN restricts its independent diagnostic effectiveness. As a result, additional research is required to determine standardized thresholds and investigate its potential as a therapeutic target for preventing tubular inflammation and fibrosis related to CIN.

#### 4.6.3. Hepcidin

Hepcidin serves as a crucial regulator of iron homeostasis and has been identified as a potential biomarker for kidney injury. Hepcidin, synthesized by the liver, regulates iron absorption and distribution [140]. Its levels are affected by systemic inflammation and oxidative stress, which are integral to the pathophysiology of CIN. Hepcidin levels can be quantified in serum or urine through ELISA or mass spectrometry techniques. Several studies indicate that hepcidin levels in urine and serum may serve as predictors for the risk of AKI development. Moreover, hepcidin has been studied as a potential biomarker in patients undergoing elective PCI. A study conducted by Malyszko et al. demonstrated that serum hepcidin levels rise significantly within 4 to 8 h following the administration of contrast media, whereas urinary hepcidin levels exhibit a notable decrease at 8 and 24 h in patients who experience CIN. This indicates that hepcidin could be significant in the early identification of CIN, facilitating prompt risk evaluation and intervention. The inverse correlation between serum and urinary hepcidin levels in patients undergoing PCI suggests its potential utility as an early predictive biomarker for CIN. The increase in serum hepcidin and the decrease in urinary hepcidin levels may indicate early indicators of kidney injury [141,142,143,144,145]. Further large-scale studies are necessary to validate its predictive value across various stages of CKD and enhance early risk stratification in clinical practice.

#### 4.6.4. Midkine

Midkine is a heparin-binding growth factor involved in inflammation, angiogenesis, and cellular repair processes. In the kidney, midkine is primarily expressed in proximal and distal tubular epithelial cells. During CIN, contrast media cause medullary hypoxia and oxidative stress, leading to the production of midkine by renal tubular cells. Increased midkine concentrations in serum and urine indicate inflammation and tissue damage, serving as a significant marker for the detection of renal dysfunction [146,147,148]. Midkine levels can be quantified through immunoassays, including ELISA, and increased concentrations are linked to oxidative stress and renal inflammation.

A study conducted by J. Malyszko et al. assessed 89 PCI patients with normal sCr levels, revealing that midkine levels rose significantly as early as 2 h post-PCI (*p* < 0.001) and remained elevated at 4 and 8 h, normalizing after 24 h. The study revealed that patients who developed CIN (10% prevalence) exhibited significantly higher midkine levels and received a greater contrast volume (*p* < 0.05). This suggests that midkine may act as a rapid and sensitive predictor of renal ischemia or nephrotoxicity in CIN [149].

In a similar study, Ahmed et al. examined 100 CKD patients (eGFR < 60 mL/min/1.73 m^2^) who underwent PCI for acute coronary syndrome (ACS) and reported that 27% experienced CI-AKI. Patients exhibited a history of diabetes, dyslipidemia, diuretic or metformin usage, and a low left ventricular ejection fraction (LVEF < 45%). Serum midkine levels measured 2 h post-PCI were significantly elevated in the CI-AKI group, and ROC curve analysis validated its predictive capacity for CI-AKI [150].

The results indicate that midkine serves as a potential early biomarker for the detection of CIN, especially in high-risk PCI patients. Given the narrow therapeutic window of CIN, the early detection of midkine may facilitate timely interventions and nephroprotective strategies to mitigate additional kidney damage.

### 4.7. Fibrosis and Long-Term Kidney Damage Biomarkers

#### 4.7.1. Connective Tissue Growth Factor (CTGF)

Connective Tissue Growth Factor (CTGF) is a profibrotic cytokine integral to tissue remodeling and extracellular matrix deposition. The expression of this biomarker is increased in response to renal tubular damage and inflammation, indicating its potential utility for the detection and monitoring of CIN [151,152,153].

In CIN, oxidative stress and inflammation triggered by contrast media may promote the release of CTGF. Increased levels of CTGF are associated with fibrosis and scarring, potentially resulting in chronic renal dysfunction. Although primarily linked to chronic processes, CTGF may also offer insights into the progression of CIN beyond the acute phase. CTGF is essential in kidney disease and fibrosis, primarily by facilitating tubular cell hypertrophy and epithelial–mesenchymal transition (EMT) in tubular epithelial cells (TECs). Elevated CTGF expression is linked to tubulointerstitial fibrosis, a significant characteristic of advancing kidney disease [153]. Zheng et al. conducted a study indicating that aldosterone-induced kidney fibrosis in mice resulted in increased CTGF levels, thereby facilitating disease progression. Treatment with miR-26a-enriched exosomes significantly decreased fibrosis through the inhibition of CTGF expression. Exosomes safeguarded miR-26a from degradation and facilitated its transport to the kidney, resulting in diminished EMT and suppression of the CTGF/SMAD signaling pathway. This indicates that targeting CTGF through miR-26a therapy may represent an effective approach to reduce kidney fibrosis and its related complications [152].

CTGF is identified in serum, urine, or renal tissues through ELISA or immunohistochemistry techniques [12,154]. Elevated CTGF levels suggest ongoing fibrosis; however, they lack specificity for CIN and may also be present in conditions like diabetic nephropathy or CKD. CTGF serves as a late marker of injury, thereby diminishing its utility for the early detection of CIN. Notwithstanding these limitations, incorporating CTGF measurements into long-term monitoring protocols enables clinicians to more effectively assess the progression of CIN and implement strategies to reduce renal fibrosis.

#### 4.7.2. Uromodulin

Uromodulin, or Tamm–Horsfall protein, represents the predominant protein excreted in urine under standard physiological conditions. Renal tubular cells in the thick ascending limb of the loop of Henle secrete this substance, which plays protective roles by inhibiting crystal formation and preventing urinary tract infections. Numerous studies and statements provide evidence that uromodulin levels in urine and serum may function as a marker for monitoring kidney function and detecting AKI [155,156,157,158].

In the context of CIN, reduced uromodulin levels after administration of contrast media may signify tubular dysfunction and increased vulnerability to injury. During CIN, oxidative stress induced by contrast media and medullary hypoxia hinder uromodulin secretion, indicating damage to tubular epithelial cells. Decreased uromodulin concentrations in urine or serum may serve as an early indicator of tubular dysfunction. A study conducted by Erdem Çankaya et al. examined the short-term alterations in serum and urine uromodulin levels prior to and following the administration of iodinated contrast agents in 86 subjects with normal renal function. The findings indicated no significant alterations in GFR following contrast administration. However, serum uromodulin levels exhibited a significant decrease (from 43.4 ± 17.6 to 24.8 ± 17.9, *p* ≤ 0.05), whereas urinary uromodulin levels demonstrated a significant increase (from 670 ± 175 to 805 ± 340, *p* ≤ 0.05). These findings indicate that serum uromodulin may function as a biomarker for ischemic kidney injury or nephrotoxic effects, whereas urinary uromodulin could offer insights into renal susceptibility to nephrotoxic agents, potentially facilitating the early detection of kidney stress or injury after contrast exposure [159]. Nonetheless, its diagnostic utility is constrained by the lack of standardized cutoff values and variability influenced by factors including hydration status and sample handling.

Uromodulin is commonly quantified through ELISA or Western blot methodologies. Although not exclusive to CIN and reducible in other tubular disorders, it offers significant insights into tubular health and injury. The integration of uromodulin measurements into a comprehensive biomarker panel could improve the early identification and management of CIN. Understanding the role of uromodulin in renal protection enables clinicians to investigate potential therapeutic strategies aimed at reducing tubular injury in CIN.

### 4.8. Biomarkers Related to Metabolic and Systemic Risk Factors

#### 4.8.1. Uric Acid (UA)

Serum UA levels are assessed via standard blood tests, with normal reference ranges generally established as 3.4–7.0 mg/dL for men and 2.4–6.0 mg/dL for women. A 24 h urine test can be conducted to evaluate UA excretion. Elevated sUA levels have been studied as potential risk factors for CIN across diverse patient populations, including individuals with diabetes, hypertension, and chronic kidney disease. Numerous clinical studies have demonstrated a significant correlation between increased UA levels and the risk of CIN [12,160,161,162,163,164,165]. Research conducted by Barbieri et al. and Mandurino-Mirizzi et al. indicated that patients with sUA levels of ≥7 mg/dL and ≥6.7 mg/dL, respectively, exhibited a markedly increased risk of developing CIN [161,162]. Tang et al. identified a cutoff of 425.5 µmol/L (~7.15 mg/dL) as an independent predictor of CI-AKI in diabetic patients undergoing CA [163]. Saylık et al. demonstrated that the serum UA-to-albumin ratio (UAR) serves as a superior predictor of CIN compared to UA alone, with a cutoff value of 1.62 achieving 87.4% specificity [164]. Tian Zuo et al. established that hyperuricemia is independently linked to CIN and elevates in-hospital mortality rates [165].

The pathophysiological role of UA in CIN encompasses multiple mechanisms, notably the inhibition of nitric oxide, resulting in decreased nitric oxide availability, vasoconstriction, and compromised renal perfusion. Increased UA levels activate endothelin-1 and the renin–angiotensin system, worsening renal ischemia. UA also induces oxidative stress through the generation of ROS and the release of inflammatory cytokines, which further exacerbates tubular damage. Urate crystal deposition in renal tubules may result in obstructive nephropathy, exacerbating kidney injury in severe instances.

UA is an independent risk factor for CIN, remaining significant even after controlling for confounding variables including age, diabetes, and baseline renal function. Elevated UA levels are associated with a higher risk of in-hospital mortality and an increased need for dialysis. Predictive models that include UA, BUN, sCr, and HbA1C demonstrate significant accuracy in assessing the risk of CIN. Incorporating sUA levels into CIN prediction models could improve the early identification of high-risk patients, especially those undergoing PCI following STEMI. Incorporating UA levels into CIN risk assessments may enhance clinical decision-making due to its significant prognostic value. Additional studies are necessary to assess the potential of urate-lowering therapies in reducing CIN risk and enhancing patient outcomes.

#### 4.8.2. Contrast Media Volume to Creatinine Clearance Ratio (V/CrCl)

The volume of contrast media to creatinine clearance ratio (V/CrCl) has become a significant metric that incorporates both contrast dosage and renal function, providing a more tailored method for assessing the risk of CIN. Normalizing contrast volume to the patient’s renal clearance capacity, V/CrCl offers a pharmacokinetic perspective on contrast exposure, thereby serving as a valuable parameter for guiding contrast administration. Numerous studies have examined the predictive and prognostic significance of V/CrCl, providing insights into optimal cutoff values and its impact on patient outcomes [24,166,167,168,169].

A meta-analysis conducted by Nie Y et al. revealed a significant association between an elevated V/CrCl ratio and CIN, with an OR of 2.67 (95% CI: 1.88–3.78, *p* < 0.001), underscoring its predictive capability [24]. Additionally, research across diverse populations has indicated differing cutoff values for the risk of CIN. Tan N et al. established a V/CrCl threshold of 2.62 as an independent predictor of CIN, indicating a twofold increased risk when this threshold is exceeded [166]. Liu Y et al. identified a cutoff of 2.44, which retained significance after adjusting for hydration volume, indicating that hydration status affects the safe contrast limit [167]. Nie Z et al. highlighted the significance of the contrast volume to glomerular filtration rate (CV/GFR), suggesting a cutoff value of 1.78 for optimal predictive accuracy [168]. Laskey WK et al. validated V/CrCl as a pharmacokinetic marker, demonstrating that a ratio ≥3.7 correlates with an abnormal early increase in sCr following PCI [169].

In addition to its predictive utility, V/CrCl possesses prognostic significance in evaluating long-term renal outcomes and mortality risk. Liu Y et al. found that a V/CrCl greater than 2.44 correlates with an increased mortality risk, underscoring its significance in informing contrast volume choices during PCI [167]. The differences in reported cutoff values among studies indicate that patient-specific factors, such as baseline renal function and hydration status, must be taken into account when utilizing V/CrCl in clinical practice. Despite variations among studies, there is a general agreement that V/CrCl serves as a reliable and independent predictor of CIN. Future research should focus on establishing standardized thresholds and incorporating V/CrCl into risk models to enhance contrast administration strategies and improve patient outcomes.

#### 4.8.3. Pre-Procedural Hyperglycemia

Pre-procedural hyperglycemia, characterized by elevated blood glucose levels prior to CAG or PCI or contrast agent administration before CT, is increasingly acknowledged as a notable risk factor for CIN. Glucose levels may be assessed through fasting blood glucose (FBG), random blood glucose (RBG), or glycosylated hemoglobin (HbA1c). Pre-procedural hyperglycemia is typically evaluated at the time of hospital admission or immediately prior to contrast administration.

Various studies have employed different cutoff values to define hyperglycemia, typically ≥140 mg/dL or ≥200 mg/dL, with elevated levels associated with an increased risk of CIN [170,171,172,173,174,175]. Kewcharoen et al. conducted a meta-analysis indicating that hyperglycemic patients face a heightened risk of CIN, regardless of their diabetic status, with pooled OR between 1.71 and 2.07 based on the glucose threshold applied [170]. Stolker et al. highlighted that pre-procedural hyperglycemia significantly correlates with CIN in non-diabetic patients, demonstrating a graded increase in risk as glucose levels exceed 110 mg/dL [171]. Lin et al. established that hyperglycemia (>198 mg/dL) serves as an independent predictor of CIN and long-term mortality in patients undergoing emergency PCI [173]. Additionally, elevated HbA1c levels in diabetic patients undergoing CAG/PCI were associated with an increased risk of CIN, especially when levels surpassed 9.5%, as reported in the study by Zhang et al. [172]. The prognostic significance of pre-procedural hyperglycemia encompasses more than just the risk of CIN. Research conducted by Li et al. and Shan et al. emphasizes the importance of the stress hyperglycemia ratio (SHR) in forecasting both CIN and negative cardiovascular events. SHR, defined as the ratio of admission blood glucose to the estimated average glucose from HbA1c, exhibited a reverse J-shaped relationship with CIN, suggesting that both excessively high and low values are associated with increased renal risk [174,175].

Despite its clinical significance, several limitations are present, including variability in the timing of glucose measurements, absence of standardized cutoff values, and confounding factors such as acute stress responses, insulin resistance, and underlying comorbidities. Routine assessment of pre-procedural glucose, ideally conducted at admission and immediately prior to contrast exposure, may assist in identifying high-risk patients due to its significant impact. Future research should investigate the potential of targeted glucose control strategies to reduce the risk of CIN and enhance patient outcomes in both diabetic and non-diabetic populations.

#### 4.8.4. Hypoalbuminemia

Hypoalbuminemia is characterized by serum albumin levels that are generally less than 3.5 g/dL. Serum albumin, synthesized by the liver, is essential for maintaining oncotic pressure, facilitating the transport of hormones, drugs, and various substances, and providing antioxidant effects. This measurement is obtained via a blood test, typically conducted during routine blood panels or assessments of liver function.

Numerous studies, such as a systematic review conducted by Liu L et al. and research by Wang D et al., have established hypoalbuminemia as an independent risk factor for CIN. A serum albumin cutoff value of <4.0 g/dL has been established to predict the risk of CIN, although specific thresholds may vary based on population and context [176,177,178,179,180,181,182,183,184].

Hypoalbuminemia is associated with an elevated risk of developing CIN and correlates with poorer outcomes in affected patients. Numerous studies indicate that patients exhibiting lower albumin levels experience elevated rates of major adverse cardiac events, extended ICU and hospital durations, and heightened mortality rates. The anti-inflammatory and antioxidant properties of albumin likely contribute to the protection of vascular and renal endothelium, thereby decreasing the risk of oxidative stress-related damage during contrast exposure [179].

Hypoalbuminemia serves as a valuable marker; however, it possesses certain limitations. Serum albumin levels are affected by various factors beyond renal function, such as nutritional status, liver function, inflammation, and fluid status. Consequently, low albumin may not solely indicate susceptibility to CIN but rather a complex interaction of multiple physiological stresses. Hypoalbuminemia does not offer direct insight into the mechanisms underlying kidney injury; instead, it correlates with systemic susceptibility to injury.

Monitoring serum albumin levels is crucial for risk stratification in CIN, especially among at-risk populations undergoing procedures involving contrast media. Due to its modifiable characteristics, managing hypoalbuminemia via nutritional or medical interventions may serve as a viable strategy for decreasing the risk of CIN; however, further research is required to substantiate this method.

### 4.9. Emerging Biomarkers with Potential in CIN

#### 4.9.1. Renalase

Renalase is an approximately 38 kDa flavoprotein that is primarily secreted by the kidneys, specifically by tubule epithelial cells. It acts as a catecholamine-metabolizing amine oxidase, which is essential for the regulation of blood pressure. Renalase is present in multiple renal structures, such as tubule epithelial cells, glomeruli, proximal and distal tubules, mesangial cells, and podocytes. It exhibits cytokine and anomerase activity, which plays a role in maintaining cardiovascular and renal homeostasis. Synthesized primarily by proximal tubular cells, it is released into the bloodstream and urine, playing a role in the regulation of catecholamine metabolism and the maintenance of vascular stability [185,186]. Its function is closely associated with renal perfusion and systemic hemodynamic stability, positioning it as a potential biomarker for the detection and management of CIN.

Contrast media-induced tubular injury and medullary hypoxia during CIN result in decreased renalase secretion. This reduction hinders the metabolism of catecholamines, leading to increased sympathetic activity and vasoconstriction. Hemodynamic instability leads to increased renal ischemia, thereby advancing the progression of CIN. A study by Zhao et al. demonstrated that renalase mitigates the detrimental effects of contrast media while also maintaining renal physiology via protective mechanisms. In their rat model of ioversol-induced CI-AKI, pretreatment with recombinant renalase (2 mg/kg) markedly enhanced renal function and diminished tubular necrosis, oxidative stress, apoptosis, and inflammation. Furthermore, renalase provided protection to HK2 cells from contrast-induced cytotoxicity through the inhibition of Caspase-3 activity and oxidative stress. Furthermore, urinary renalase levels were observed to be decreased in the group undergoing coronary angiography (CA) or percutaneous coronary intervention (PCI), indicating a potential role for renalase as a biomarker and therapeutic target in the prevention of CIN [187,188].

Renalase concentrations can be quantified in serum or urine through ELISA or mass spectrometry techniques [12]. Decreased renalase levels correlate with kidney dysfunction; however, a universally accepted cutoff for predicting CIN remains undefined. The diagnostic utility is further complicated by systemic conditions, including hypertension, diabetes, and cardiovascular diseases, which may also affect renalase levels.

Notwithstanding these limitations, renalase presents distinct advantages as a biomarker. This not only indicates renal function but also offers insights into systemic hemodynamic regulation, positioning it as a potential therapeutic target in CIN. Further research is required to determine standardized cutoff values and investigate their clinical applications. Integrating renalase measurements with additional biomarkers may provide clinicians with a more thorough understanding of the pathophysiology of CIN, thereby enhancing prevention and treatment strategies.

#### 4.9.2. Brain Natriuretic Peptide (BNP)

Brain Natriuretic Peptide (BNP) is a cardiac hormone secreted predominantly by ventricular cardiomyocytes in response to myocardial stretch and elevated ventricular pressure. BNP, commonly linked to heart failure and cardiovascular issues, is pertinent to renal injury due to its capacity to indicate haemodynamic alterations and fluid overload, both of which are significant risk factors for renal injury.

Recently, BNP has emerged as a significant predictive biomarker and a possible protective agent for CIN [189,190,191,192,193]. Iodinated contrast media can worsen fluid retention, renal vasoconstriction, and hemodynamic instability during CIN, especially in patients with pre-existing cardiovascular conditions. Increased BNP levels may reflect underlying stress, acting as an indirect predictor of CIN risk. BNP levels exceeding 100 pg/mL are correlated with a heightened risk of CIN, especially in individuals undergoing PCI [194].

Numerous studies have examined the role of BNP in the prevention of CI-AKI. A randomized controlled trial conducted by Jinming Liu et al. investigated the effects of BNP administration in patients with CKD undergoing PCI or CAG. The patients were randomly assigned to receive either BNP infusion (0.005 µg/kg/min) in conjunction with saline hydration or saline hydration alone. The findings indicated that BNP significantly decreased the incidence of CI-AKI (6.6% compared to 16.5%, *p* = 0.025) and maintained renal function, as evidenced by reduced sCr and CysC levels, along with a more stable eGFR over time [189]. A subsequent larger study conducted by the same research group, which included 1000 patients diagnosed with unstable angina, provided additional confirmation of the renoprotective effects of BNP. Patients administered BNP prior to contrast exposure exhibited significantly reduced incidence of CI-AKI (5.6% vs. 14.8%, *p* < 0.01) and demonstrated more rapid recovery of renal function in comparison to those who received solely saline hydration. The findings indicate that recombinant human BNP (rhBNP) could be an effective therapeutic approach to reduce renal damage induced by cardiomyopathy (CM) [190].

A meta-analysis by Xuefeng Wu et al. evaluated 12 studies with 7789 patients, revealing that BNP exhibited a pooled sensitivity of 0.73 and a specificity of 0.77 for predicting CI-AKI, alongside an area under the receiver operating characteristic (ROC) curve of 0.80. The findings indicate that BNP may function as an early detection mechanism for CI-AKI, enhancing risk stratification among high-risk patients [191]. A study conducted by Rudolf Jarai et al. analyzed BNP levels in 979 patients with ST-segment elevation myocardial infarction (STEMI) who underwent PCI, utilizing data from the HORIZONS-AMI trial. Baseline BNP levels showed a significant association with the development of CI-AKI, even after controlling for clinical and laboratory variables (*p* < 0.001). The incorporation of BNP into risk prediction models, including the Mehran Risk Score, enhanced the precision of patient risk stratification [192].

A meta-analysis by Xiaoming Li et al. in 2020 further supports the diagnostic potential of BNP by evaluating the predictive value of BNP and NT-proBNP in patients with ACS undergoing CAG. The analysis encompassed nine trials involving 2832 patients, yielding a pooled sensitivity of 0.73 and specificity of 0.79. These findings indicate that BNP and NT-proBNP serve as effective markers for the early detection of CI-AKI. The study highlighted the necessity for additional research to establish optimal cutoff values and evaluate the advantages of integrating BNP with other biomarkers to enhance diagnostic accuracy [193].

BNP has shown significant sensitivity and specificity as a predictive marker for identifying high-risk patients undergoing contrast-based procedures. BNP administration prior to contrast media exposure demonstrates significant renoprotective effects, especially in patients with CKD or ACS. Although these findings are encouraging, additional large-scale, multi-center clinical trials are required to determine standardized BNP cutoff values, enhance its application in clinical practice, and confirm its effectiveness in preventing CIN.

#### 4.9.3. Gamma-Glutamyl Transferase (GGT)

Gamma-glutamyl transferase (GGT) is an enzyme that is predominantly located in the liver, pancreas, and kidney tissues, playing a significant role in glutathione metabolism and antioxidant defense mechanisms. GGT is found in the brush border of the renal proximal tubules and is excreted in the urine (uGGT) following tubular injury [195]. Research indicates that oxidative stress, endothelial vascular injury, and inflammation—key factors in CIN—may be associated with increased GGT levels, positioning it as a potential biomarker for predicting and monitoring CIN progression [196,197].

Initial clinical studies revealed a notable elevation in uGGT activity after the administration of contrast media, suggesting tubular toxicity. Donadio et al. conducted a comparison of GGT and alanine aminopeptidase (AAP) in a cohort of 49 renal patients receiving radiological procedures involving contrast media administration [197]. AAP and GGT levels exhibited a significant increase following contrast exposure, peaking on the first day and maintaining elevated levels for several days thereafter. The concordance among these enzymes was significant, with GGT exhibiting greater repeatability, thereby reinforcing its role as a marker for CM-induced tubular damage [198].

A study conducted by Fatih Oksuz examined 473 patients with STEMI who underwent primary PCI (PPCI), further supporting the predictive role of GGT. Patients were categorized into tertiles according to baseline GGT levels, revealing a significantly elevated incidence of CIN in the highest GGT tertile (>33 U/L) compared to the lower two tertiles (29% vs. 11%, *p* < 0.001). Furthermore, in-hospital mortality rose across tertiles. ROC analysis determined a GGT threshold of >26.5 U/L for predicting CIN, achieving a sensitivity of 70% and a specificity of 60%. Multivariate regression analysis revealed that diabetes mellitus, C-reactive protein levels, contrast volume, and elevated GGT serve as independent predictors of CIN. Each 1 U/L increase in GGT was significantly associated with an elevated risk of CIN (*p* < 0.001), underscoring its prognostic relevance [93].

GGT’s routine availability in clinical practice is a notable strength, as it is frequently included in liver function tests. GGT levels are generally assessed through enzymatic assays; however, a specific cutoff for predicting CIN has not been defined. Elevated GGT levels require interpretation alongside additional clinical findings and biomarkers. The findings indicate that GGT serves as a significant biomarker for the detection and prediction of CIN, providing a non-invasive approach for early risk stratification. Current evidence underscores its potential; however, additional large-scale studies are necessary to confirm optimal cutoff values and evaluate its clinical integration with other biomarkers for enhanced CIN diagnosis and prevention.

#### 4.9.4. MicroRNAs (miRNAs)

MicroRNAs (miRNAs) are increasingly recognized as significant biomarkers for the early identification of kidney injury [199,200,201,202,203,204]. Small, non-coding RNA molecules play a crucial role in regulating gene expression at the post-transcriptional level and are implicated in various cellular processes, including apoptosis, inflammation, and oxidative stress.

The stability of miRNAs in biological fluids, including serum and urine, represents a significant advantage, rendering them appropriate for non-invasive testing. Advanced molecular techniques such as quantitative PCR (qPCR), microarrays and next-generation sequencing (NGS) facilitate accurate profiling of miRNA expression. These methods are characterized by complexity, high costs, and the necessity for specialized expertise, thereby restricting their routine application in clinical practice. Moreover, systemic conditions like diabetes, cardiovascular disease, and inflammation can affect miRNA levels, thereby diminishing their specificity for CIN [205,206].

MiR-30a, miR-30e, miR-188, and miR-21 demonstrate considerable potential as biomarkers for the early diagnosis and prognosis of CIN. MiR-30a and miR-188 are linked through the MAPK-JNK/p38 signaling cascade, which facilitates apoptosis induced by contrast media. Research indicates that elevated levels of miR-30a (fold change of 1.405) and miR-30e (fold change of 1.428) are associated with a higher incidence of CIN, exhibiting AUC values of 0.802 and 0.805, respectively. miR-188 exhibited a fold change exceeding 1.343, resulting in an AUC of 0.784, indicating high specificity (93.0%) and moderate sensitivity (52.1%). The elevated levels of these miRNAs result in a specificity of 97.2%, indicating their potential as robust diagnostic markers for CIN. Their stability in serum and urine renders them appropriate for early detection and monitoring [202].

miR-21 is a notable microRNA associated with AKI and CIN. It is essential in regulating apoptosis, inflammation, and fibrosis in kidney injury. Increased levels of miR-21 have been noted following renal ischemia/reperfusion injury and are linked to kidney cell proliferation and decreased apoptosis. Within the framework of CIN, miR-21 has been investigated for its predictive significance in the progression of AKI. Clinical studies indicate that elevated urinary and plasma levels of miR-21 are associated with the severity of AKI, demonstrating an area under the curve (AUC) of 0.81 in urine and 0.83 in plasma. Additionally, miR-21 levels serve as predictors for the necessity of renal replacement therapy, AKIN stage 3 acute kidney injury, and extended hospitalization in patients undergoing cardiac surgery. The findings indicate that miR-21 may function as a prognostic marker for AKI, particularly in cases related to CIN [203,204].

MiR-30a, miR-30e, miR-188, and miR-21 demonstrate considerable potential as biomarkers for the early diagnosis and prognosis of CIN. Their role in critical molecular pathways associated with kidney injury underscores their importance for early intervention and potential therapeutic targeting in patients susceptible to contrast-induced renal damage.

## 5. Evolving Strategies and Challenges in CIN Prevention and Management

The incorporation of biomarkers into clinical practice for the prevention and management of CIN represents a promising strategy that has the potential to improve patient outcomes through timely intervention.

One of the key advantages of early biomarker detection is the opportunity to apply preventive strategies like hydration protocols, which are essential in minimizing the risk of CIN. Research indicates that both oral and intravenous hydration administered prior to and following contrast exposure can markedly reduce the incidence of CIN, especially among high-risk patients [4,207,208].

Moreover, biomarkers facilitate the early detection of patients who are at risk, empowering clinicians to adjust or eliminate nephrotoxic agents, which further reduces the potential for renal injury [5]. A notable benefit of biomarker integration is its ability to enable customized interventions that are in accordance with the principles of personalized medicine, enhancing the application of nephroprotective strategies and minimizing CIN-related morbidity, leading to improved renal outcomes and minimizing the necessity for dialysis or extended hospital stays.

While there are promising advantages to biomarker-based strategies, numerous obstacles need to be overcome prior to their broad application in clinical settings. A significant constraint is the absence of standardized protocols and validation among various populations, which obstructs the uniform application of biomarker findings. Furthermore, the substantial expense associated with biomarker testing can create a financial obstacle, especially in healthcare environments with limited resources, thereby restricting broad accessibility [209,210].

Moreover, effective incorporation of biomarkers into standard CIN management necessitates targeted education and training for healthcare practitioners. Grasping the interpretation of biomarker data and their integration into clinical decision-making is essential for enhancing patient care. It is essential to establish guidelines and training programs that empower clinicians to effectively use biomarkers in the prevention and treatment of CIN [211].

This review highlights biomarkers; however, it is crucial to acknowledge that contrast media may lead to additional adverse effects beyond CIN. Contrast agents can present potential risks, including allergic reactions, hemodynamic instability, and issues associated with cumulative exposure during repeated imaging studies [212,213]. Furthermore, the application of contrast media in pediatric populations introduces distinct challenges, given that younger patients may exhibit varying renal sensitivities and metabolic reactions in comparison to adults [214,215]. Future investigations should not solely concentrate on biomarkers but also take into account these wider issues, guaranteeing a thorough approach to contrast media safety across various patient groups.

## 6. Future Directions and Emerging Innovations in CIN Management

Advanced technologies and computational analysis are being utilized to discover new biomarkers that can more accurately predict kidney injury compared to conventional approaches [216,217]. Machine learning (ML) tools are essential for analyzing intricate datasets, such as mass spectrometry and genomic sequencing data, to identify significant features and categorize potential biomarkers [217,218]. Furthermore, computational bioinformatics models are incorporating molecular networks and cross-level biomarkers to enhance the understanding of CIN pathogenesis and facilitate biomarker identification [216].

The integration of biomarker data with clinical information is revolutionizing the prediction of CIN risk through the application of artificial intelligence (AI) and ML. These technologies examine extensive datasets to uncover risk patterns that might not be visible through conventional statistical approaches, enhancing early detection and patient stratification (232). AI systems are being created to integrate omics data with clinical parameters, facilitating personalized risk assessments and customized interventions [219,220]. Moreover, the analysis of digital biomarkers using deep learning techniques is becoming a promising non-invasive method for assessing the risk of CIN. The biomarkers obtained from imaging and sensor data provide a comprehensive and evolving view of patient health, thereby improving decision-making in clinical environments [220].

The incorporation of AI-driven biomarker analysis presents considerable opportunities for enhancing the management of CIN. Enhancing diagnostic accuracy, refining risk assessment, and tailoring treatment approaches can significantly decrease the occurrence of CIN and improve patient outcomes. Nonetheless, additional investigation is required to confirm these methods across varied patient groups and to tackle issues concerning data standardization, clinical application, and accessibility.

## 7. Conclusions

Biomarker-based approaches present considerable potential in the management of CIN. However, addressing challenges associated with cost, standardization, and clinical implementation is essential for their broader acceptance. Future investigations ought to concentrate on confirming biomarkers in more extensive patient cohorts, creating economical testing techniques, and improving educational programs for healthcare professionals. Addressing these barriers could facilitate the integration of biomarkers, leading to more effective strategies for preventing CIN and ultimately reducing renal complications and enhancing patient outcomes.

## Figures and Tables

**Table 1:** Biomarkers for CIN based on different features, advantages, and limitations.

Biomarker	Location	Sample (Blood/Urine)	Type of Marker	Advantages	Disadvantages	Cutoff for CI-AKI Prediction	Detection Methods	**Other Considerations**
**Traditional Markers of Kidney Function**								
Serum Creatinine (sCr)	Glomerulus	Blood	Diagnostic	Widely used, standardized	Late indicator of injury	≥0.5 mg/dL or ≥25% increase from baseline within 48–72 h	Enzymatic assay, colorimetric assay	Influenced by muscle mass, hydration
Glomerular Filtration Rate (GFR)	Glomerulus (flow of plasma from the glomerulus into Bowman’s space)	Blood	Diagnostic	Reflects kidney function	Indirect measurement	eGFR < 60 mL/min/1.73 m^2^	MDRD, CKD-EPI equation	Requires age, sex, and race adjustments
Urinary Output (UO)	Tubules	Urine	Diagnostic	Easy to monitor	Requires catheterization	<0.5 mL/kg/h for >6 h	Urine collection	Variable due to hydration status
Proteinuria and Microalbuminuria	Glomerulus and tubules	Urine	Diagnostic	Early marker of kidney stress	Nonspecific	Urine protein > 150 mg/day	Dipstick, spot urine test, 24 h urine collection	Can be influenced by hypertension, diabetes
**Glomerular Filtration and Tubular Dysfunction Biomarkers**								
Cystatin C (CysC)	Glomerulus	Blood	Predictive	Early detection, less influenced by muscle mass	Affected by inflammation	>15% increase within 24–48 h	Immunoassay, nephelometry, turbidimetry	Thyroid dysfunction, inflammation, or corticosteroid use might increase its levels
Beta-2 Microglobulin (β2M)	Glomerulus proximal tubules	Blood, urine	Diagnostic	Sensitive to tubular dysfunction	Influenced by systemic diseases	Increased urinary β2M, with >1.26 mg/L at baseline	Immunoassay, ELISA	Elevated in multiple myeloma, infections
Retinol-Binding Protein (RBP)	Glomerulus proximal tubules	Blood, urine	Predictive	Early marker of tubular injury	Affected by liver function	Not available	ELISA, nephelometry	Not specific to CIN, can be elevated in other conditions such as diabetes, obesity, coronavirus disease, and malnutrition
Vitamin D Binding Protein (VDBP)	Glomerulus proximal tubules	Urine	Predictive	Predicts long-term kidney damage	Expensive testing	Urine VDBP > 613 ng/mL	ELISA	Linked to CKD progression, potential to serve as a predictor of dialysis use
**Tubular Injury Biomarkers**								
Kidney Injury Molecule-1 (KIM-1)	Proximal tubules	Blood, urine	Diagnostic	Highly specific for tubular injury	Limited routine use	Urine KIM-1 > 3× baseline; ranging from >0.048 to 6.33 ng/mL	ELISA, immunoassay	Useful for early CIN detection
Neutrophil Gelatinase-Associated Lipocalin (NGAL)	Glomerulus proximal and distal tubules	Blood, urine	Predictive	Early detection within hours	Nonspecific (also elevated in sepsis)	Plasma/urine NGAL > 150 ng/mL	ELISA, point-of-care tests	Increases within 2–6 h post-exposure
N-Acetyl-β-D-Glucosaminidase (NAG)	Proximal tubules	Urine	Predictive	Sensitive to tubular damage	Not CIN-specific	Increased NAG activity	ELISA, enzymatic assay, fluorometry	Elevated in diabetes, hypertension, and other clinical conditions
Liver Fatty Acid-Binding Protein (L-FABP)	Proximal tubules	Urine	Predictive	Early tubular hypoxia marker	Limited specificity	Peak at 12 h post-contrast ≥24.5 μg/g	ELISA	Also elevated in CKD, CVD, and various non-CIN conditions
α-GST/π-GST	Proximal and distal tubules	Urine	Predictive	Site-specific renal injury	Limited clinical adoption	Increased levels of α-GST indicate proximal injury, while π-GST indicates distal injury	ELISA	Used in AKI prognosis; α-GST: proximal injury, π-GST: distal injury
Clusterin	Proximal and distal tubules	Urine	Predictive	Early nephrotoxicity marker	Lacks specificity and less studied in CIN	Early rise post-contrast	ELISA, Western blot	Linked to tissue repair
**Inflammatory and Oxidative Stress Biomarkers**								
Interleukin-18 (IL-18)	Proximal and distal tubules	Urine	Predictive	Early AKI marker	Affected by systemic inflammation	Urine IL-18 > 25% at 24 h	ELISA, Bioplex	Peaks at 24 h, declines at 72 h
C-Reactive Protein (CRP) and high-sensitivity C-Reactive Protein (hs-CRP)	Systemic inflammation	Blood	Predictive	Readily available	Nonspecific	CRP levels > 3.0 mg/L and hs-CRP levels > 5 mg/d exhibited a significantly elevated risk of CIN	Immunoassay	Unspecific inflammatory biomarkers
Neutrophil-to-Lymphocyte Ratio (NLR)	Systemic	Blood	Predictive	Inexpensive inflammatory marker	Not kidney-specific	Increased in CIN	CBC count	Emerging biomarker for systemic inflammation, might provide prognostic significance in cardiovascular diseases and renal dysfunction
Red Cell Distribution Width (RDW)	Systemic	Blood	Predictive	Predicts CIN risk	Affected by anemia, nutrition	Elevated in CIN patients	CBC count	Emerging biomarker
**Cell Stress and Apoptosis Biomarkers**								
IGFBP-7 and TIMP-2	Renal epithelial cells Proximal tubules	Urine	Predictive	Predicts AKI risk	Expensive assay	>0.3 (ng/mL)^2^/1000 for the [IGFBP-7] × [TIMP-2] product	NephroCheck^®^	Detects subclinical AKI
Dickkopf-3 (DKK3)	Renal epithelial cells	Urine	Predictive	Early fibrosis predictor	Under investigation	Increased in CKD patients	ELISA	Linked to CKD progression
Growth Differentiation Factor-15 (GDF-15)	Tubules	Blood	Predictive	Potential prognostic marker	Limited studies	Elevated in CIN	ELISA	Associated with CKD progression
**Vascular and Endothelial Dysfunction Biomarkers**								
Vascular Endothelial Growth Factor (VEGF)	Endothelium	Blood, urine	Predictive	Role in vascular repair	Lacks specificity and insufficient current clinical data on CIN	Increased in CIN	ELISA	Target for nephroprotection
Osteopontin (OPN)	Proximal and distal tubules Loop of Henle	Blood, urine	Predictive	Predicts renal inflammation	Affected by CKD, diabetes	Elevated in CIN	ELISA, Western blot	Part of multi-marker models Limited study about CIN
Hepcidin	Tubules	Blood, urine	Predictive	Iron regulation and kidney injury marker	Limited clinical use	Post-contrast Serum ↓, urine ↑ hepcidin	ELISA	Potential predictive biomarker
Midkine (MK)	Proximal and distal tubules	Blood, urine	Predictive	Early ischemia marker	Needs more validation	Early rise about 2 h post-contrast	ELISA	Related to oxidative stress
**Fibrosis and Long-Term Kidney Damage Biomarkers**								
Connective Tissue Growth Factor (CTGF)	Tubules	Blood, urine	Predictive	Chronic injury marker	Late-stage marker	Increased in CIN	ELISA, IHC	Linked to fibrosis progression
Uromodulin	Renal tubular cells in the thick ascending limb of the loop of Henle	Blood, urine	Predictive	Protective biomarker	Not CIN-specific	Post-contrast serum ↓, urine ↑ uromodulin	ELISA, Western blot	May predict nephrotoxicity
**Biomarkers Related to Metabolic and Systemic Risk Factors**								
Uric Acid (UA)	Glomerulus	Blood	Predictive	Associated with CKD progression	Affected by diet, hydration	sUA levels of ≥7 mg/dL may increase risk of developing CIN	Uric acid test (enzymatic)	High levels linked to oxidative stress
Contrast media volume to creatinine clearance ratio (V/CrCl)	Systemic	Blood	Predictive	Predicts CIN risk pre-procedure	Requires creatinine clearance calculation	V/CrCl > 3.7 as high risk	Mathematical calculation	Affected by hydration status
Pre-procedural hyperglycemia	Systemic	Blood	Predictive	Predicts CIN in diabetic patients	Not kidney-specific	>180 mg/dL linked to CIN	Blood glucose test	Linked to oxidative stress and inflammation
Hypoalbuminemia	Systemic	Blood	Predictive	Marker of malnutrition and inflammation	Affected by liver disease, critical illness	<3.5 g/dL linked to CIN	Serum albumin test	Low levels indicate poor prognosis
**Emerging Biomarkers with Potential in CIN**								
Renalase	Proximal tubules	Blood, urine	Predictive	Cardiovascular–renal link	Lacks standardization	Lower in CIN	ELISA, mass spectrometry	Potential therapeutic target
Brain Natriuretic Peptide (BNP)	Systemic	Blood	Predictive	Predicts CIN risk	Affected by heart failure	BNP > 100 pg/mL	Immunoassay	Used in volume status assessment
Gamma-Glutamyl Transferase (GGT)	Systemic	Blood	Predictive	Oxidative stress marker	Affected by liver disease	>26.5 U/L for predicting CIN	Enzymatic assay	Linked to CKD progression
MicroRNAs (miRNAs)	Tubular cells	Blood, urine	Predictive	Early molecular marker	Expensive, requires expertise	miR-30a, miR-21 elevated in CIN	qPCR, NGS	Promising in precision medicine
